# Cholangioscopy-assisted endoscopic mucosal resection for a mildly dysplastic lesion of the common bile duct: a pilot exploration for super minimally invasive surgery

**DOI:** 10.1055/a-2257-3531

**Published:** 2024-02-15

**Authors:** Wengang Zhang, Ningli Chai, Yujie Feng, Jiafeng Wang, Qingzhen Wu, Bo Zhang, Enqiang Linghu

**Affiliations:** 1 651943 Gastroenterology, Chinese PLA General Hospital First Medical Center, Beijing, China; 2 104607 Gastroenterology, Chinese PLA General Hospital, Beijing, China

A 61-year-old man underwent endoscopic retrograde cholangiopancreatography (ERCP) for common bile duct (CBD) stones in our hospital. We performed peroral cholangioscopy to confirm whether any remnant stones were left after basket extraction; however, a polypoid lesion with a wide base was found in the CBD ( Fig. 1 **a** ). We performed biopsy under cholangioscopic guidance, with pathology results revealing mild dysplasia. This patient faced a dilemma: surgical treatment would be accompanied by relatively major trauma; however, follow-up observation carries a risk of lesion progression. Therefore, our team developed a kind of injection needle and a snare with electrocision function, which can be passed through the working channel of the cholangioscope. Subsequently, we confirmed the safety and feasibility of cholangioscopy-assisted endoscopic mucosal resection (CA-EMR) in the CBD in a porcine model 1 . In this study, we performed CA-EMR for the aforementioned patient and successfully resected the CBD lesion.

**Fig. 1:**
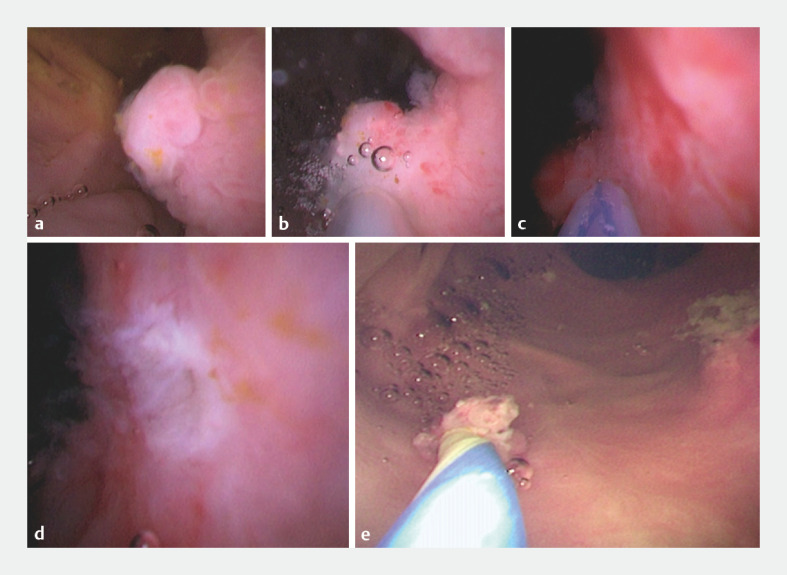
Cholangioscopic images showing: **a** a polypoid lesion with a wide base, which was found in the middle of the common bile duct; **b** the lesion after submucosal injection was performed at the base of the lesion using an injection needle under direct vision; **c** the polypoid lesion being successfully resected using the snare with its electrocision function; **d** the pale appearance of the subsequent wound; **e** the polypoid lesion being extracted using a basket under direct vision.

First, a cholangioscope with a 1.8-mm working channel (EyeMax, 11 Fr; Micro-Tech) was inserted into the CBD. Second, submucosal injection was performed at the base of the polypoid lesion using the injection needle under direct vision ( Fig. 1 **b** ). Third, the specially designed snare was inserted into the CBD through the working channel of the cholangioscope. Fourth, the polypoid lesion was resected successfully using the snare, by its electrocision function, with the wound showing a pale appearance ( Fig. 1 **c,d** ). Fifth, the polypoid lesion was extracted from the body using a basket under direct vision ( Fig. 1 **e** ). Finally, endoscopic nasobiliary drainage was performed ( Video 1 ). The postoperative pathology results showed mild dysplasia. The patient’s recovery was smooth, with no adverse events detected on the basis of abdominal signs and computed tomography, following 1 month of follow-up.

The cholangioscopy-assisted endoscopic mucosal resection (CA-EMR) procedure is performed for an area of mild dysplasia in the common bile duct.Video 1

With the popularization of radiological techniques, more and more polypoid lesions are being found in the biliary duct system 2 3 . This study offered preliminary confirmation that CA-EMR could be used to resect polypoid lesions of the CBD accurately, avoiding surgical treatment, and may be of benefit in patients with polypoid lesions in the biliary duct system.

Endoscopy_UCTN_Code_TTT_1AR_2AD

## Notes

Endoscopy E-Videos https://eref.thieme.de/e-videos
*E-Videos* is an open access online section of the journal *Endoscopy* , reporting on interesting cases and new techniques in gastroenterological endoscopy. All papers include a high-quality video and are published with a Creative Commons CC-BY license. Endoscopy E-Videos qualify for HINARI discounts and waivers and eligibility is automatically checked during the submission process. We grant 100% waivers to articles whose corresponding authors are based in Group A countries and 50% waivers to those who are based in Group B countries as classified by Research4Life (see: https://www.research4life.org/access/eligibility/ ). This section has its own submission website at https://mc.manuscriptcentral.com/e-videos .
